# Preeclamptic heart failure — perioperative concerns and management: a narrative review

**DOI:** 10.1186/s13741-024-00391-x

**Published:** 2024-05-10

**Authors:** Anjishnujit Bandyopadhyay, Sunaakshi Puri, Tanvir Samra, Vighnesh Ashok

**Affiliations:** 1 Department of Anaesthesiology, Pain Medicine and Critical Care, JPNATC, All India Institute of Medical Science, New Delhi, India; 2 Department of Paediatric Anaesthesia, Post Graduate Institute of Child Health, Noida, India; 3 Department of Anaesthesia and Intensive Care, Post Graduate Institute of Medical Education and Research, Chandigarh, 160012 India

**Keywords:** Cesarean section, Heart failure, Labor, Obstetrics, Preeclampsia

## Abstract

Preeclampsia is an important cause of heart failure during pregnancy and the postpartum period. The aim of this review is to elucidate the pathophysiology and clinical features of preeclamptic heart failure and describe the medical and anesthetic management of these high-risk parturients. This article reviews the current evidence base regarding preeclamptic heart failure and its pathophysiology, types, and clinical features. We also describe the medical and anesthetic management of these patients during the peripartum period. Heart failure due to preeclampsia can present as either systolic or diastolic dysfunction. The management strategies of systolic heart failure include dietary salt restriction, diuresis, and cautious use of beta-blockers and vasodilators. Diuretics are the mainstay in the treatment of diastolic heart failure. In the absence of obstetric indications, vaginal delivery is the safest mode of delivery in these high-risk patients, and the use of an early labor epidural for analgesia is recommended. These patients would require increased invasive monitoring during labor and vaginal delivery. Neuraxial and general anesthesia have been used successfully for cesarean section in these patients but require crucial modifications of the standard technique. Uterotonic drugs have significant cardiovascular and pulmonary effects, and a clear understanding of these is essential during the management of these patients. Preeclamptics with heart failure require individualized peripartum care, as cardiac decompensation is an important risk factor for maternal and neonatal morbidity and mortality. These high-risk parturients benefit from timely multidisciplinary team inputs and collaborated management.

## Introduction

Hypertensive disorders in pregnancy are an important cause of maternal morbidity and mortality. In addition to being an important risk factor for long-term maternal cardiovascular disease, it also contributes to a significant risk of fetal prematurity and mortality (Mehta et al. 2020). With a significant variation in racial and geographic distribution, hypertensive disorders in pregnancy disproportionately affect women of Black, African-American, and Alaskan Native women (Umesawa and Kobashi 2017). Hypertensive disorders in pregnancy comprise gestational hypertension, preeclampsia (with and without severe features), preeclampsia superimposed on chronic hypertension, and eclampsia (Rana et al. 2019; Say et al. 2014).

Cardiovascular diseases complicate up to 4% of all pregnancies with the incidence likely to be higher if hypertensive disorders are also included (Regitz-Zagrosek et al. 2018). Among all the cardiovascular diseases complicating pregnancy, heart failure (HF) remains the most common complication, regardless of the cause, and the incidence has been increasing over the past decades (Stergiopoulos et al. 2019; Mogos et al. 2018). Preeclampsia is an important risk factor for the development of HF during pregnancy and the postpartum period (Anthony and Sliwa 2016). The prevalence of HF in preeclamptic parturients is relatively underexplored and underreported probably owing to the challenges in accurate diagnosis and the resulting misclassifications. Preeclamptic parturients can develop HF of two distinct types. While some preeclamptics develop a form of HF characterized by left ventricular (LV) systolic dysfunction, referred to as HF with reduced ejection fraction (HFrEF), others exhibit variable degrees of LV diastolic dysfunction, resulting in HF with preserved ejection fraction (HFpEF) (Bright et al. 2021). The pathophysiology, cardiovascular mechanics, therapeutic options, and peripartum management strategies are different for these two forms of HF in preeclamptic parturients. It is, hence, crucial that clinicians be able to effectively screen for HF, correctly identify the sub-type, and risk-stratify preeclamptic parturients with HF, so that appropriate targeted interventions can then be initiated during the peripartum period. This review attempts to summarize the pathophysiology of preeclamptic HF, followed by a detailed description of the current evidence base and clinical considerations regarding the peripartum anesthetic and critical care management of preeclamptic parturients with HF.

## Cardiovascular changes in pregnancy and preeclampsia

During the course of a normal pregnancy, the maternal cardiovascular system undergoes extensive adaptations in order to maintain an adequate uteroplacental perfusion throughout pregnancy (Ramlakhan et al. 2020). By 8 weeks of gestational age, maternal systemic vascular resistance decreases by 10–30% and reaches a nadir between 20th and 26th weeks of pregnancy. This decline in SVR is accompanied by a decline in mean arterial pressure, which reverses from 26th to 28th weeks, reaching prepregnancy values at full term (Ouzounian and Elkayam 2012; Duvekot et al. 1993). The inotropic and chronotropic effects of pregnancy occur secondary to the effect of stimulation of baroreceptors in the cardiopulmonary and renal vasculature, with a subsequent increase in the contractility and heart rate by 15–25% and stroke volume by 20–30%. This brings about an attendant increase in cardiac output by 30–50%. Anatomically, the increase in preload and myocardial contractility manifests in LV hypertrophy and a larger left atrial diameter (Hunter and Robson 1992; Savu et al. 2012; Meah et al. 2016; Gaasch and Zile 2011). These cardiovascular changes are usually benign and fully recover within 1 year of delivery (Gaasch and Zile 2011; Melchiorre et al. 2016).

Preeclampsia results from the failure of embryonic cytotrophoblasts to invade and remodel the myometrial spiral arteries (Rana et al. 2019). The resultant placental bed comprises of arteries that are small and hyper-responsive to vasomotor stimuli, resulting in a mismatch between uteroplacental supply and fetal demands (Burton et al. 2019). This sets off a cascade of pro-inflammatory responses and endothelial dysfunction in the maternal circulation leading to increased maternal systemic vascular resistance (Orabona et al. 2018). This “arterial stiffness,” and the subsequent lower plasma volume expansion and reduced cardiac output, triggers maternal neurohormonal hyperactivity culminating in a vicious cycle of sustained maternal hypertension (Orabona et al. 2018). While these observations are consistent in untreated preeclamptics, they are contrasting to results in treated preeclamptics, who present with more heterogeneous cardiovascular variables. Furthermore, the pattern and degree of cardiac remodeling also seem to be influenced by the time of onset of preeclampsia (early onset vs late onset). In preeclamptics, the LV remodeling presents as concentric LV hypertrophy leading to left ventricular systolic dysfunction, impaired myocardial contractility, and a reduced LV ejection fraction, resulting in HFrEF (Melchiorre et al. 2014; Castleman et al. 2016). LV diastolic dysfunction, on the other hand, characterized by reduced regional myocardial relaxation, is a direct consequence of myocardial stiffening due to LV hypertrophy, which is reflected in left atrial remodeling by means of elevated left-sided filling pressure (Rafik Hamad et al. 2009; Melchiorre et al. 2011; Ponikowski et al. 2016a). About 50% of patients develop myocyte loss, subsequent fibrosis, and LV remodeling resulting in HFrEF, manifesting as reduced pump efficiency and impaired peripheral perfusion (Metra and Teerlink 2017). On the other hand, the pathophysiology of HFpEF is still not clear, but it seems to be related to a pro-inflammatory state resulting in endothelial dysfunction, myocyte hypertrophy, and collagen deposition together with fibrosis and reduced LV compliance. Ineffective calcium metabolism within the sarcoplasmic reticulum and altered mitochondrial density within the myocardium have also been implicated in the pathophysiology of diastolic dysfunction in preeclamptics (Maaten et al. 2016).

However, not all preeclamptics with features of maladaptive cardiac remodeling during pregnancy develop acute cardiac failure (Melchiorre et al. 2014). Acute decompensation of the cardiovascular system could occur in the presence of one or more precipitating factors, resulting in overt HF (Table 1).

**Table 1:** Precipitating factors for heart failure in preeclamptics

**Cardiac factors**	**Hemodynamic factors**	**Intravascular fluid status**	**Others**
• Myocardial ischemia • Atrial and ventricular arrhythmias	• Increased intravascular hydrostatic pressure (acute hypertension) • Increased endothelial permeability • Decrease in intravascular oncotic pressure (albumin loss, postpartum autotransfusion)	• Autotransfusion after delivery • Iatrogenic fluid overload • Uterotonics (oxytocin)	• Pain • Anxiety • Labor • Anesthesia

## Clinical features

Preeclamptic parturients present with hypertension after 20 weeks of gestation along with new onset proteinuria, with or without severe features. Severe features include blurring of vision, headache, seizures (eclampsia), dyspnea/hypoxia, right upper quadrant pain, nausea/vomiting, oligohydramnios, intrauterine growth restriction, and in some cases multiorgan dysfunction (Burton et al. 2019). In addition to the abovementioned spectrum of symptoms, a preeclamptic parturient in HF could present with sudden onset dyspnea, orthopnea, paroxysmal nocturnal dyspnea, increased fatigue, and syncopal attacks. Clinical examination could reveal tachycardia, pedal edema, elevated jugular venous pressures, hypo/hypertension, displacement of the apical impulse, audible third heart sound, and variable degree of pulmonary crepitation (Anthony and Sliwa 2016; Jensen et al. 2007). Several of these clinical features can occur as part of the cardiovascular and respiratory physiological changes that occur with normal pregnancy. Hence, a high degree of clinical suspicion and a low threshold for diagnostic investigations are warranted. 

The differential diagnosis of obstetric acute HF is quite wide, and a clear understanding of these various entities is essential for an obstetric anesthesiologist (Table 2). An important and distinct cause of HFrEF in the peripartum period is peripartum cardiomyopathy (PPCM) and is a diagnosis by exclusion (Bauersachs et al. 2019).

**Table 2:** Differential diagnosis of obstetric acute heart failure

**Diagnosis**	**Clinical presentation**	**Role of echocardiography**
Acute coronary syndrome	• Typical chest pain with localizing ECG changes • Arrhythmias, heart block • Elevated troponin levels	• Identification of regional wall motion abnormalities • Detect complications of myocardial infarction: acute mitral regurgitation, ventricular septal rupture, ventricular wall rupture, and cardiac tamponade
Arrhythmias	• Palpitations • Syncopal attacks	• Could help rule out structural defects predisposing to arrhythmias (e.g., valvular disease) • Rule out the presence of thrombus in heart chambers
Valvulopathies	• Worsening of preexisting symptoms in known heart disease patients • New onset dyspnea, orthopnea, paroxysmal nocturnal dyspnea, and palpitations	• Detect structural valvular/septal defects • Pulmonary arterial hypertension • Chamber enlargement • Rule out intracardiac thrombi
Preeclampsia	• New-onset hypertension after 20 weeks’ gestation associated with proteinuria, adverse conditions, or multi-organ dysfunction	• Identify and grade LV concentric hypertrophy • Detect and quantify LV systolic and diastolic dysfunction
Peripartum cardiomyopathy	• HF toward the end of pregnancy or postpartum • Diagnosis of exclusion • Elevated natriuretic peptide levels	• LV systolic dysfunction and ejection faction < 45% • Global hypokinesia
Takotsubo cardiomyopathy	• Ischemic-like chest pain and transient ECG changes • Elevated troponin level	• Identify characteristic apical ballooning with free wall-sparing
Septic cardiomyopathy	• Systemic features of sepsis • Hypo/hyperthermia • Hypotension • Raised inflammatory markers	• Demonstrate global hypokinesia with a hyperdynamic circulation

*ECG* electrocardiogram, *LV* left ventricle

## Investigational workup

Increased total leukocyte count, low platelets, elevated liver enzymes, serum creatinine, and uric acid are part of the spectrum of preeclampsia (Burton et al. 2019). While CKMB levels are unreliable during pregnancy and labor, cardiac-specific troponin levels are generally elevated in preeclamptics even in the absence of overt HF, leading to diagnostic uncertainty (Dockree et al. 2021). Recently, studies have evaluated the role of brain natriuretic peptide (BNP) and N-terminal pro-BNP (NT-BNP) in the diagnosis and prognostication of HF in obstetrics. BNP and NT-BNP are peptides released by the atria and ventricles in response to increased wall tension. They remain normal throughout pregnancy but are mildly elevated in preeclampsia (Kampman et al. 2014). However, they are markedly increased in PPCM and preeclamptic HF (Sliwa et al. 2010). A value of *BNP* < 100 pg/ml and *NT-proBNP* < 300 pg/ml usually rules out HF. An electrocardiogram is recommended in all preeclamptics with suspected HF. The presence of arrhythmias, ST-T segment changes, or block patterns could help in diagnosing the etiology and complications of HF (Ramlakhan et al. 2020). The presence of B lines on lung ultrasonogram is also suggestive of pulmonary edema or fluid overload with HF (Muniz et al. 2018; Maw et al. 2019).

Echocardiography involves the ultrasonographic assessment of cardiac structure and function, and transthoracic (TTE) has been increasingly used in the perioperative management of sick parturients. Echocardiography is particularly useful in the differential diagnosis of acute HF symptomatology (Table 2). A “focused TTE,” also known as “goal-directed TTE,” is an abridged version of a more comprehensive 2-dimensional TTE, and it is a point of care and noninvasive bedside tool. The main aim of this focused study is the prompt recognition of gross abnormalities using the standard echocardiographic views, i.e., parasternal, subcostal, and apical, so as to facilitate early stabilization and institution of treatment (Griffiths et al. 2018).

The Rapid Obstetric Screening Echocardiography (ROSE) is an obstetric-specific focused TTE, which takes into consideration the anatomic and physiological changes occurring during pregnancy (Dennis 2011). It comprises of the basic TTE views to identify gross abnormalities, but could also include a more detailed assessment of cardiac output using Doppler and an assessment of diastolic function by more experienced clinicians. The basic principles of the ROSE scan are described in Table 3. A detailed review of a comprehensive echocardiographic examination is beyond the scope of this article and can be found elsewhere (Castleman et al. 2016; Rafik Hamad et al. 2009).

**Table 3:** Principles of the Rapid Obstetric Screening Echocardiography (ROSE) scan (Dennis 2011)

A	*A*cceptable and *A*pplicable as it is safe and non-invasive
B	Can be performed *B*edside of the patient (point of care)
C	*C*oncise examination (parasternal and apical views) and *C*omfortable for parturients
D	*D*iagnose contractility disorders and hypo/hypervolemia and treat accordingly
E	*E*mbolus (clot, amniotic fluid, and air), assessment of right heart function, and structure
F	*F*etal heart rate monitoring by experienced clinicians

## Management of HF in preeclampsia

In view of the paucity of research performed specifically in preeclamptic parturients with HF, most of the recommendations regarding clinical management are derived from clinical studies on HF in non-obstetric patients or expert opinion (Mehta et al. 2020; Regitz-Zagrosek et al. 2018). There exists a broad consensus that treatment modalities should avoid adverse fetal effects in parturients, and treating clinicians must be cognizant of the fetal safety profile of drugs used for the management of symptoms of HF in women who are pregnant and breastfeeding (Table 4). Obstetric patients with heart failure are best managed in a high dependency or critical care unit due to increased morbidity and mortality.

**Table 4:** Safety profile of drugs used in preeclamptic HF during pregnancy and lactation

**Drug**	**Use in pregnancy**	**Use during lactation**	**Potential adverse effects**
Loop diuretics (furosemide)	Compatible	Compatible	Maternal hypovolemia and hypotension, uterine hypoperfusion, decreased breast milk production
Beta-blockers	Compatible	Compatible	Fetal hypoglycemia, fetal bradycardia
Nitrates (glyceryl trinitrate)	Compatible	Compatible	Maternal hypotension, uterine hypoperfusion
ACE inhibitors/ARBs	Incompatible	Compatible	Renal agenesis, oligohydramnios, fetal loss
Mineralocorticoid receptor antagonists	Incompatible	Compatible	Fetal undervirilization
Digoxin	Compatible	Compatible	Low birth weight
Heparin (UFH, LMWH)	Compatible	Compatible	Increased maternal bleeding risk, implication on timing of neuraxial interventions
Warfarin	Avoid	Compatible	Fetal skeletal deformities, intracranial bleeding, abortion, and stillbirth

*UFH* unfractionated heparin, *LMWH* low molecular weight heparin, *ACE* angiotensin-converting enzyme, *ARB* angiotensin receptor blocker

## HF with reduced ejection fraction

### Initial pharmacotherapy

While the European and American guidelines recommend sodium restriction as the first step in the management of volume status in HF, its role in an acute hospital setting is limited (Mehta et al. 2020; Regitz-Zagrosek et al. 2018). Loop diuretics could be added for symptomatic pulmonary or peripheral edema, while taking special care to avoid over-diuresis, which could result in maternal hypotension, uterine hypoperfusion, and fetal hypoxia (Hilfiker-Kleiner D et al. 2015; Maaten et al. 2015). Calcium channel blockers are useful and safe for the treatment of hypertension and arrhythmias in these patients. Cardio-selective beta-blockers (e.g., metoprolol) are safe but recommended only if maternal hemodynamics are stable. Drugs commonly used to prevent cardiac remodeling like angiotensin-converting enzyme inhibitors, angiotensin receptor blockers, and mineralocorticoid receptor antagonists are contraindicated due to their potential teratogenicity. In hypertensive pulmonary edema, vasodilators like intravenous hydralazine and nitroglycerine (glyceryl trinitride) are safe and recommended (Ponikowski et al. 2016b; Irani and Xia 2008).

### Vasopressors and inotropes

Inotropic support may be needed in acutely ill parturients with severely impaired ventricular function. There is, however, very limited data regarding the use, safety, and long-term outcomes with the use of inotropes in preeclamptic heart failure. Phenylephrine and norepinephrine can be used safely to maintain the blood pressure in hypotensive parturients in cardiogenic shock. In cases of severe low cardiac output, inotropes like dopamine, dobutamine, or milrinone may be needed and can be started cautiously, with an aim to wean the patient from them as early as hemodynamically possible. The use of beta-agonists may be detrimental in HF due to PPCM. Levosimendan, as a calcium-sensitizing agent rather than a sympathomimetic drug, could have a role as an inotropic agent in preeclamptic HF (Stapel et al. 2017; Labbene et al. 2017).

### Mechanical circulatory support and ventricular assist devices

In patients with severe cardiogenic shock not responding to pharmacotherapy, mechanical circulatory support devices like intra-aortic balloon pump and venoarterial extracorporeal membrane oxygenation have been used as a bridge to recovery or cardiac transplantation (Gevaert et al. 2011). However, preeclamptic HF could resolve within 6 months postpartum, which must be considered to guide any clinical decision-making regarding the implantation of long-term devices, viz., ventricular assist devices (Zimmerman et al. 2010).

### Bromocriptine

It has been demonstrated that increased oxidative stress during the late gestation and early postpartum period causes cleavage of prolactin (23 kDa) to a prolactin fragment called vasoinhibin (16 kDa). Vasoinhibin has strong pro-inflammatory, antiangiogenic, and pro-apoptotic in vivo, which restricts oxygen and nutrient supply to the heart by disrupting the blood vessels, resulting in HF (Koenig et al. 2018). This concept prompted research using bromocriptine to inhibit prolactin secretion, thus limiting the damage to the heart in PPCM. The current literature evidence regarding the use of bromocriptine in PPCM is conflicting (Sliwa et al. 2010; Yaméogo et al. 2017; Haghikia et al. 2013; Hilfiker-Kleiner et al. 2017). Until more definitive results are available, bromocriptine should be considered experimental. Because of its effect on lactation suppression, the implications of using bromocriptine in the postpartum period should be discussed with the patient, and the decision individualized. Currently, the 2018 European Society of Cardiology guidelines include a weak recommendation (class IIb, level of evidence: B) for the use of bromocriptine in obstetric HF due to PPCM (Regitz-Zagrosek et al. 2018). Due to the association of bromocriptine with thrombotic complications, therapeutic anticoagulation is recommended when its use is being considered.

### Magnesium

Intravenous or intramuscular magnesium sulfate is the first-line seizure prophylaxis in preeclamptic with severe features. Multiple reviews have established its superiority over other pharmacotherapeutic agents in this regard (Gestational hypertension and preeclampsia 2020). But its use is not without attendant risks of respiratory and cardiac depression, more so in patients with compromised cardiac function. Magnesium sulfate is a potent vasodilator and has a significant negative inotropic and dromotropic effect on the heart. While these effects are usually well tolerated in healthy individuals, magnesium might have significant implications if the cardiovascular system is already compromised (Allen and Magnesium 2023). In the absence of clear-cut evidence or consensus-based guidelines, regarding the usage and dosage of magnesium sulfate in patients with preeclamptic HF, the decision to use magnesium sulfate in such cohort of patients has to be a multidisciplinary one after meticulous weighing in of all the risks and the proposed benefits. A lesser dose with more frequent serum magnesium level and clinical monitoring might be prudent.

## HF with preserved ejection fraction

In contrast to treatment of HFrEF, there are even fewer studies done on patients with HFpEF. Since most studies of HFpEF have been performed on elder patients with more complex cardiac comorbidities including coronary artery disease, it is challenging to extrapolate these data onto preeclamptic obstetric patients with HFpEF. Currently, diuretics are the only class of drugs that are recommended for treating fluid overload in parturients with HFpEF, keeping in mind the fetal effects of the drugs. There are currently no recommendations regarding the use of other groups of drugs for the treatment of HFpEF in preeclamptic parturients (Henning 2020; Deshmukh et al. 2020).

## Arrhythmia management

Preeclamptics with HF are at a risk of cardiac dysrhythmias, with atrial fibrillation being the most common arrhythmia. Cardio-selective beta-blockers, digoxin, and calcium channel blockers are safe and effective antiarrhythmics for use in pregnancy (Joglar and Page 1999). In unstable patients, cardioversion/defibrillation, wherever applicable, is safe and should be performed without any delay. The decision to perform cardioversion/defibrillation under intravenous sedation or after securing the airway with an endotracheal tube must be taken with due consideration to the fasting status of the patient and the potential risk of gastric regurgitation and pulmonary aspiration (Ferrero et al. 2004). Whenever possible, it may be prudent to use fetal monitoring during cardioversion/defibrillation, due to the theoretical risk of inducing secondary arrhythmia in the fetus.

Ventricular dysfunction is an important risk factor for ventricular tachyarrhythmias and sudden cardiac death. However, given the higher rate of recovery of LV function with delivery and drug therapy, the early implantation of implantable cardioverter-defibrillators is generally not recommended. Wearable cardioverter-defibrillators have been proposed as a bridge to full cardiac recovery (Kober et al. 2016; Elming et al. 2017; Duncker et al. 2017).

## Anticoagulation

Pregnancy is a procoagulant state, and HF and atrial fibrillation are independent risk factor for systemic thromboembolism (Dunkman et al. 1993; Refuerzo et al. 2003). The increased procoagulant state persists into the postpartum period, making these patients susceptible to peripheral arterial and venous thromboembolism. In this context, prophylactic administration of unfractionated or low molecular weight heparin is recommended. Patients with persistent atrial fibrillation, evidence of systemic thromboembolism, and those with an intracardiac thrombus, however, require therapeutic anticoagulation (Nelson-Piercy et al. 1997; Howie 1986).

## Planning of delivery

Termination of pregnancy is recommended in cases of severe preeclampsia and eclampsia. Multidisciplinary team input is necessary for planning for delivery, and a coordinated liaison between obstetricians, anesthesiologists, cardiologists, intensivists, and neonatologists is crucial for shared and individualized decision-making in these patients (Easter et al. 2020). Severe preeclampsia and eclampsia are maternal indications for urgent termination of pregnancy, and for a majority of parturients with HF, vaginal delivery remains the safest option, in the absence of obstetric indications for cesarean delivery (Regitz-Zagrosek et al. 2018). For vaginal delivery, it is important to note that intense and prolonged Valsalva maneuver during straining and bearing down may be poorly tolerated in preeclamptic parturients with HF. This is due to rapid reduction in preload and increase in afterload that occurs normally during the Valsalva’s maneuver. Moreover, the overshoot in the cardiac output that occurs upon release of the Valsalva could exacerbate symptoms of HF in these parturients (Nishimura and Tajik 1986, 2004). Urgent cesarean delivery regardless of the gestational age should be considered in rapidly worsening parturients with hemodynamic instability despite ongoing medical therapy for HF. In significantly high-risk parturients, a planned cesarean delivery may offer the advantage of avoiding emergent delivery permitting the availability of senior clinicians.

## Analgesia for vaginal delivery

Effective labor analgesia preserves hemodynamic stability and cardiopulmonary function during labor. By abolishing the pain-induced release of catecholamines, good neuraxial labor analgesia could attenuate the increase in myocardial oxygen demand and increased cardiac output and fluctuations in preload and afterload during labor (Shnider et al. 1983; Jouppila et al. 1982; Ramos-Santos et al. 1991). Therefore, in the absence of any contraindications, most obstetric anesthesiologists prefer to initiate epidural analgesia as early as possible in these patients. Epidural-only, combined spinal-epidural (CSE), and intrathecal opioid-only CSE techniques have all been successfully employed to improve analgesic efficacy, quality of block, and sacral root coverage (Ankichetty et al. 2013; Dennis 2012; Pascual-Ramirez et al. 2011). Whatever the technique used, it is important to gradually titrate the epidural drugs to avoid rapid reductions in systemic vascular resistance and preload, which can cause precipitous hypotension.

All preeclamptic parturients with HF must have appropriate cardiac monitoring during labor to predict and prevent serious maternal cardiac events. Waveform pulse plethysmography, electrocardiogram, and noninvasive blood pressure monitoring at regular intervals are minimum monitoring standards for these high-risk parturients. An invasive arterial line placement could be considered in sicker laboring parturients, particularly those who have a higher chance of requiring an urgent cesarean section. The indwelling arterial line could be invaluable during the rapid conversion of epidural labor analgesia into epidural anesthesia for cesarean delivery and in instances when general anesthesia is required for emergency cesarean delivery. Since laboring parturients often move and strain during the course of labor and delivery, measurement of central venous pressure is often of low clinical utility (Nishimura and Tajik 2004). However, insertion of a central venous catheter may be considered in instances where continuous infusion of vasoactive drugs is anticipated during labor and in the immediate postpartum period.

## Anesthesia for cesarean delivery

Currently, there is no consensus regarding the superiority of any single mode of anesthesia in terms of improved maternal and fetal outcomes in preeclamptic parturients with HF undergoing cesarean delivery. Regardless of the mode of anesthesia, the hemodynamic goals are common to all approaches. Sudden fall in the systemic vascular resistance should be avoided, as it can lead to maternal hypotension and uterine hypoperfusion. Preventing bradycardia and strict attention to intravenous fluid balance are other important aspects of perioperative care during operative delivery. Noninvasive cardiac output monitoring using pulse waveform analysis, suprasternal Doppler, and bioreactance have been used to guide the administration of intravenous fluids, vasopressors, and oxytocin in preeclamptics, and are valuable strategies to optimize hemodynamics of preeclamptics in HF (Armstrong et al. 2011; Ackerman-Banks et al. 2023).

Whether cesarean delivery is being performed under neuraxial or general anesthesia, an intra-arterial catheter for beat-to-beat blood pressure monitoring should be strongly considered. Additionally, in parturients requiring infusion of vasopressors/inotropes, central venous access should be established.

### Central neuraxial anesthesia

Neuraxial anesthesia, when performed well, offers the mother an opportunity to be awake and experience child birth while avoiding the complications of general anesthesia and airway instrumentation. Intrathecal local anesthetics provide a more rapid onset and dense block, as compared to epidural local anesthetics, which could reduce peripheral vascular resistance resulting in maternal hypotension. These hemodynamic responses can be attenuated with a prophylactic phenylephrine infusion. Some obstetric anesthesiologists prefer to employ a CSE technique for these patients incorporating a low-dose spinal, which can theoretically combine the reliability and predictability of a spinal anesthetic with the extendibility and hemodynamic stability of an epidural anesthetic (Hamlyn et al. 2005).

### General anesthesia

There are certain scenarios in which neuraxial anesthesia is not advisable. These include parturients on anticoagulant therapy, those who are unable to lie flat for surgery, those who require mechanical ventilation in view of worsening hemodynamics and/or pulmonary gas exchange, and in cases of severe fetal distress.

General anesthesia in healthy parturients is typically rapid sequence with no opioid premedication as part of the induction sequence (Devroe and Van d Velde M, Rex S. 2015). However, for preeclamptic parturients in HF, prioritizing maternal hemodynamic stability over risks of newborn respiratory depression with the use of opioids like fentanyl and remifentanil as part of a modified rapid sequence induction of general anesthesia may be considered (Pant et al. 2014). Remifentanil premedication at a dose of 1 mcg/kg during induction of general anesthesia in parturients has been shown to reduce the hemodynamic response to laryngoscopy, intubation, and skin incision. Although remifentanil crosses the placenta, it is rapidly cleared from the fetal circulation and is much less a respiratory depressant in the newborn, when compared to morphine and fentanyl (Heesen et al. 2013; Wadsworth et al. 2002). In view of its cardiostability, etomidate could also be used for induction of general anesthesia in high-risk parturients (Orme et al. 2004).

### Use of uterotonics

All uterotonics have significant cardiovascular effects, and a good understanding of these is important for the management of preeclamptic parturients in HF. Intravenous oxytocin remains the first-line uterotonic drug in these patients. However, the dose and rate should be carefully titrated (ideally using infusion pumps) due to its propensity to reduce systemic vascular resistance. The hypotension caused by oxytocin can be offset by carefully titrating an infusion of phenylephrine. In patients requiring postpartum oxytocin infusions, care should be taken to reduce the volume of carrier intravenous infusate, by using more concentrated oxytocin infusions, in order to avoid potential fluid overload (Langesæter et al. 2006). Intravenous carbetocin is a thermostable longer-acting analog of oxytocin and may be useful in these patients as it does not require to be given as an infusion postpartum (Pisani et al. 2012). Methylergometrine can cause vascular smooth muscle contraction, resulting in hypertension, coronary vasospasm, and myocardial ischemia (Svanstrom et al. 2008). Likewise, carboprost tromethamine can cause an increase in pulmonary arterial pressures, exacerbating right ventricular dysfunction and HF (Elbohoty et al. 2016). Methylergometrine and carboprost should be avoided in these high-risk parturients, as far as possible. Misoprostol is a weak uterotonic, which is devoid of any cardiovascular side effects. Therefore, rectal or buccal misoprostol along with carefully titrated intravenous oxytocin is a reasonable and safe option for prophylaxis of postpartum hemorrhage in these patients (Quibel et al. 2016).

## Postpartum care

The postpartum phase is a high-risk period for maternal decompensation in the case of preeclamptic patients. Regardless of the mode of delivery, the “autotransfusion” of blood from the retracting uterus and relief of pressure over the inferior vena cava can result in a significant increase in cardiac preload (Arendt and Lindley 2019). This increase in preload can place undue strain over the dysfunctional left ventricle, predisposing to the development of pulmonary edema and HF. This is particularly a problem in patients with significant diastolic dysfunction. Inadequate pain management in the immediate postpartum period can increase the heart rate, cardiac output, and maternal myocardial oxygen consumption. A combination of these factors can increase the risk of maternal cardiac decompensation in the immediate postpartum period (Melchiorre et al. 2014). Oxygen supplementation, adequate analgesia, attention to intravenous fluids, judicious use of oxytocin infusions, and cardiac monitoring are all important aspects of postpartum care in these patients.

## Obstetric critical care

An intensivist must be involved early in the peripartum management of women who are at high risk for rapid deterioration (Banerjee and Cantellow 2021a). All preeclamptic parturients who are inotrope/vasopressor dependent should be transferred early to a high dependency or critical care unit for further management, in view of their high morbidity and mortality. The specific principles of obstetric critical care for such patients are outlined in Table 5. Management specific to acute HF in pregnancy is similar to the nonpregnant state and includes optimization of preload, afterload, myocardial contractility, rhythm, maintenance of oxygenation, and organ perfusion (Banerjee and Cantellow 2021a, 2021b).

**Table 5:** Principles of obstetric critical care

**System**	**Considerations**
Respiratory support	- Noninvasive ventilation • Has been used but with caution, keeping in mind the high risk of gastric inflation, regurgitation, and aspiration - Tracheal intubation • Histamine receptor antagonist or proton-pump inhibitor in anticipation, sodium citrate solution, head-up tilt • Cricoid pressure controversial • Preoxygenation can be done using high-flow nasal cannula • Short-handled laryngoscope, ensure optimal positioning including “ramp” for patients with high BMI, and smaller-sized endotracheal tubes - Invasive mechanical ventilation • Plateau pressure — 35-cm H2O, tidal volume 6 mL/kg (ideal body weight) • Optimal PEEP titration with driving pressure ≤ 15-cm H2O • Arterial oxygen partial pressure threshold — 70 mm Hg. Consider using fetal heart rate monitor to monitor for signs of fetal hypoxia if a lower threshold is used • Maternal optimization of oxygenation and ventilation should not routinely include fetal delivery, unless fetal indications are present
Circulatory support	- In case of any hemodynamic instability, always rule out possible aorto-caval compression - Judicious use of intravenous fluids - Low threshold for initiating invasive monitoring - Evidence-based use of vasodilators, vasopressors, and inotropes - Bedside-focused transthoracic echocardiography to diagnose etiology and initiating appropriate treatment
Pharmacotherapeutic agents	- Ideal sedative is still elusive - Most sedatives will have depressant actions on the fetus, if birth is planned soon after - Opioid infusions are associated with risk of respiratory depression in the fetus - Midazolam infusions carry risk of acute fetal benzodiazepine withdrawal
Nutrition	- Most nutrition trials have excluded parturients; hence, appropriate caloric goals are unknown - Prokinetics might be needed in view of impaired gastric emptying
Prophylaxis for deep venous thrombosis	- All patients to receive prophylaxis unless otherwise contraindicated
Fetal care	- In the absence of specific maternal indication, cardiotocography is usually not a routine practice if the patient is not laboring - Obstetrician, anesthesiologist, and neonatologist must be kept informed prior and available on standby if delivery is planned in the obstetric critical care unit - Cardiotocogram, uterotonic agents, cesarean kit, and neonatal resuscitation equipment be kept ready

## Summary and conclusion

Preeclampsia is an important risk factor for the development of HF during pregnancy and postpartum, which can present as either systolic or diastolic failure. It is important for anesthesiologists to have a good understanding of the underlying pathophysiology and the current evidence base for early diagnosis and stabilization of these high-risk patients. The anesthetic management of these patients for vaginal and operative delivery is challenging and requires multidisciplinary team inputs. Early referral of these high-risk parturients to higher centers with obstetric anesthesiology, cardiology, and critical care expertise is crucial for successful obstetric and neonatal outcomes. More research is needed to develop risk stratification models, perioperative anesthesia protocols, and practice guidelines for the anesthetic and critical care management of this high-risk population subgroup.

## Data Availability

No datasets were generated or analysed during the current study.

## Abbreviations

HF: Heart failure
HFrEF: Heart failure with reduced ejection fraction
HFpEF: Heart failure with preserved ejection fraction
LV: Left ventricle
PPCM: Peripartum cardiomyopathy
BNP: Brain natriuretic peptide
NT-BNP: N-terminal pro-brain natriuretic peptide
TTE: Transthoracic echocardiography
ROSE: Rapid obstetric screening echocardiography
CSE: Combined spinal-epidural
DPE: Dural puncture epidural
ACE: Angiotensin-converting enzyme
ARB: Angiotensin receptor blocker
UFH: Unfractionated heparin
LMWH: Low molecular weight heparin
